# Innovative Use of Opsite Flexigrid^™^ for Digital Photography in Rhinoplasty

**Published:** 2013-01

**Authors:** Denis Codazzi, Maria Alessandra Bocchiotti, Bernardo Righi, Enrico Robotti

**Affiliations:** 1 Department of Plastic Surgery, University of Turin, San Giovanni Battista Hospital, Turin, Italy;; 2 Department of Plastic Surgery, Riuniti Hospital, Bergamo, Italy

**Keywords:** Opsite flexigrid, Digital Photography, Rhinoplasty

**Dear Editor**

Standardization of photography in plastic surgery is fundamental for pre-operative surgical planning, comparative post-operative assessment, and demonstration of surgical results.^1^ In rhinoplasty, slight changes in patient or camera position can lessen nasal hump, vary nose size, and alter skin tension.^2^ In order to prevent these common errors, photographic standardization with high- quality equipment (camera, lens, and lighting), consistent room set up and systematic patient position are mandatory.^2^

The authoritative Institute of Medical Illustrator^3^ published its guidelines about “Rhinoplasty and Septorhinoplasty Photography”: One of the most important concerns is about the use of standard viewfinder alignment grids to help finding both horizontal (Frankfurt and Reid planes first) and vertical reference planes during shooting. However, some cameras lack this grid at all and some other have grid with only four axes leaving focus point without reference lines. (Figure 1)

**Fig. 1:**
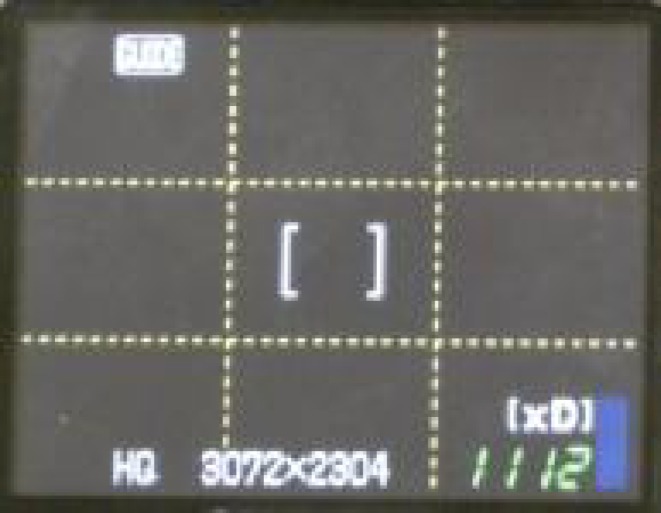
The commonest four axes grid leaves focus point without reference lines

We thought about a new application of Opsite Flexigrid^™^ (Smith and Nephew Medical Limited, Hull, HU3 2BN, England) which is a transparent, adhesive film dressing, with a measurement grid (Figure 2).

**Fig. 2:**
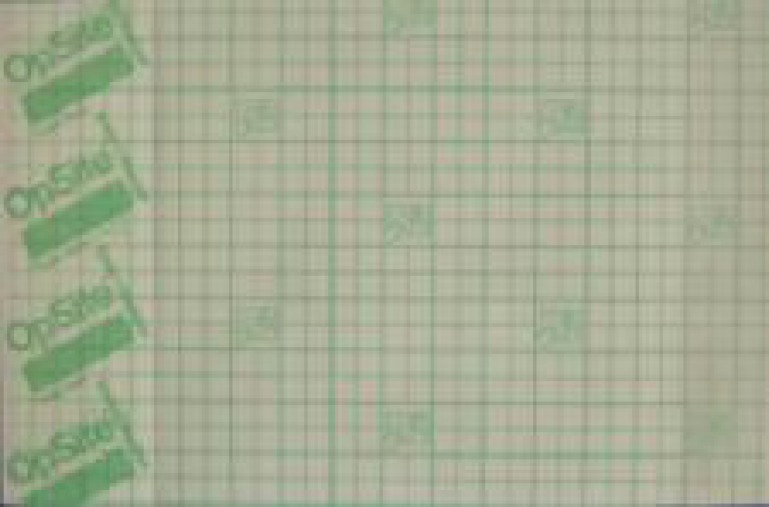
Opsite Flexigrid™ 10x12 Cm

An Opsite Flexigrid rectangle is tailored with scissors on screen camera dimensions (Figure 3).

**Fig. 3:**
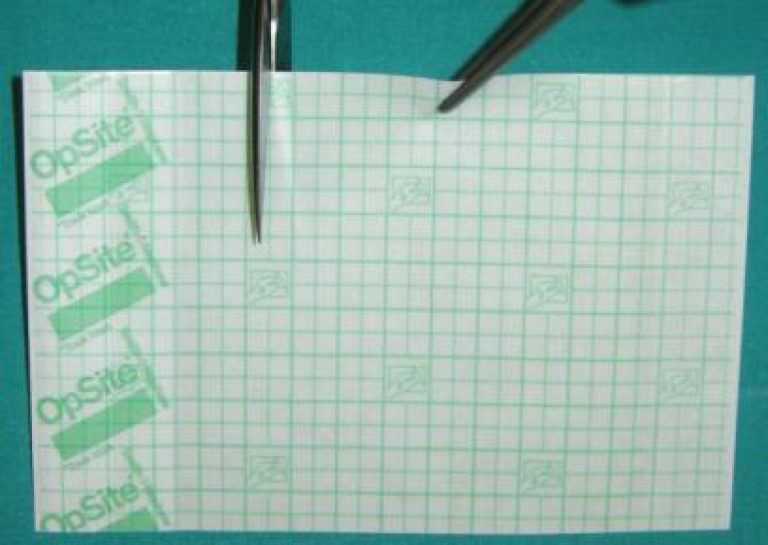
Opsite Flexigrid^™^ is tailored with scissors on screen camera dimensions

After removing the white back sheet (Figure 4), we turned on the camera and centered one of the grid intersections on camera viewfinder (Figure 5). The lines of the grid followed the main axes of the screen.

**Fig. 4:**
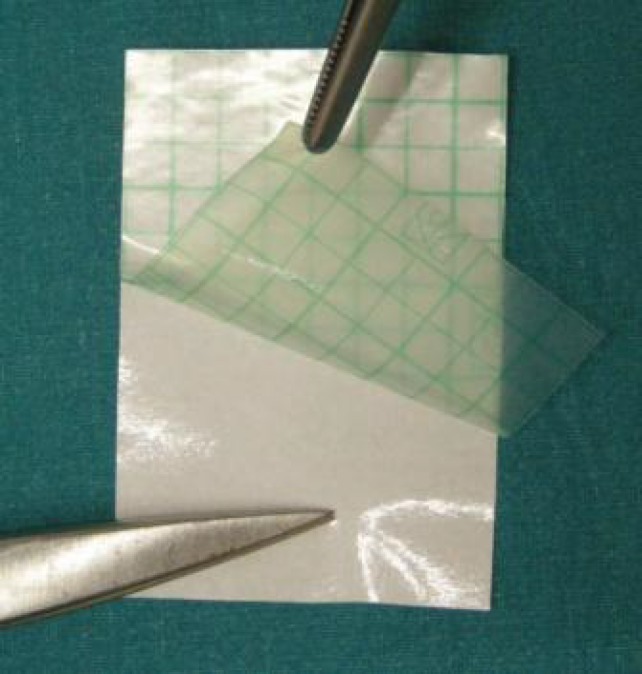
White back sheet is removed

**Fig. 5:**
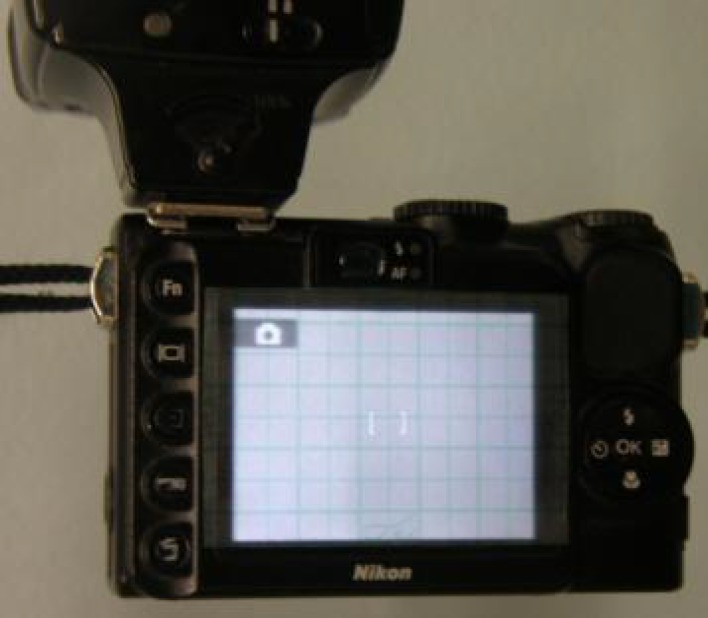
Grid is positioned on camera screen

The grid did not alter subject visibility and allowed the alignment of facial landmarks^4^in all conventional rhinoplasty pictures. This is a cheap (0.82 euro for 6x7 Cm sample, 2.04 for 10x12 Cm sample), quick and reversible way to mechanically add to digital camera a frequent lacking display option.

## CONFLICT OF INTEREST

The authors declare no conflict of interest.

## Footenote

Please cite this paper as:

Codazzi D, Bocchiotti MA, Righi B, Robotti E. Innovative Use of Opsite Flexigrid^™^ for Digital Photography in Rhinoplasty.World J PlastSurg 2013;2(1): 50-51.
